# A semi-automatic method for quantification of respiratory variation in early mitral inflow velocity using real time phase contrast cardiac magnetic resonance - normal values and clinical feasibility

**DOI:** 10.1186/1532-429X-18-S1-Q70

**Published:** 2016-01-27

**Authors:** Simon Thalén, Peder Sörensson, Andreas Sigfridsson, Martin Ugander

**Affiliations:** grid.4714.60000000419370626Department of Clinical Physiology, Karolinska Institute, Stockholm, Sweden

## Background

Echocardiography has shown that patients with constrictive pericarditis or hemodynamically significant pericardial effusion show an increased respiratory variation in early transmitral inflow velocity. The methodology for quantification of respiratory variation in early transmitral inflow velocity using real time phase contrast (RT-PC) cardiovascular magnetic resonance (CMR) images is currently cumbersome and manual. The aim of this study was to develop a method for quantifying the respiratory variation in early transmitral inflow velocity using semi-automatic analysis of RT-PC CMR images.

## Methods

Clinically referred patients (n = 25, age 55 ± 21 years, 72% male) with sinus rhythm and no pericardial effusion or pericardial thickening underwent RT-PC CMR (1.5T Siemens Aera) of short-axis through-plane transmitral inflow velocities over two 30 s acquisitions during free breathing. Image acquisition parameters were: repetition time 12 ms, water excitation pulse with flip angle 15°, slice thickness 11 mm, matrix 84 × 128, echo-planar imaging factor 7, temporal sensitivity encoding rate 3, aliasing velocity 150 cm/s, shared velocity encoding enabled and temporal resolution 60 ms. One patient with hemodynamically significant pericardial effusion was imaged to illustrate clinical feasibility. Image analysis was performed using an in-house developed plugin to Segment (Medviso AB, Lund, Sweden). The user manually delineated a region of interest encompassing the mitral orifice, and the software then automatically identified the contiguous 10 s period of time with the lowest variation in per-beat peak early transmitral inflow velocity.

## Results

Automatic analysis of RT-PC data yielded a respiratory variation in early mitral inflow velocity of mean ± SD 16 ± 5%, 95% normal limits 6-26%. The patient with pericardial effusion (20 mm) had an inflow variation of 34%.

## Conclusions

This study has demonstrated clinical feasibility and presented normal values for semi-automatic analysis of the respiratory variation in early transmitral inflow velocity using RT-PC CMR. Further studies in patients with echocardiographically verified hemodynamically significant pericardial disease are justified.

**Figure 1:**
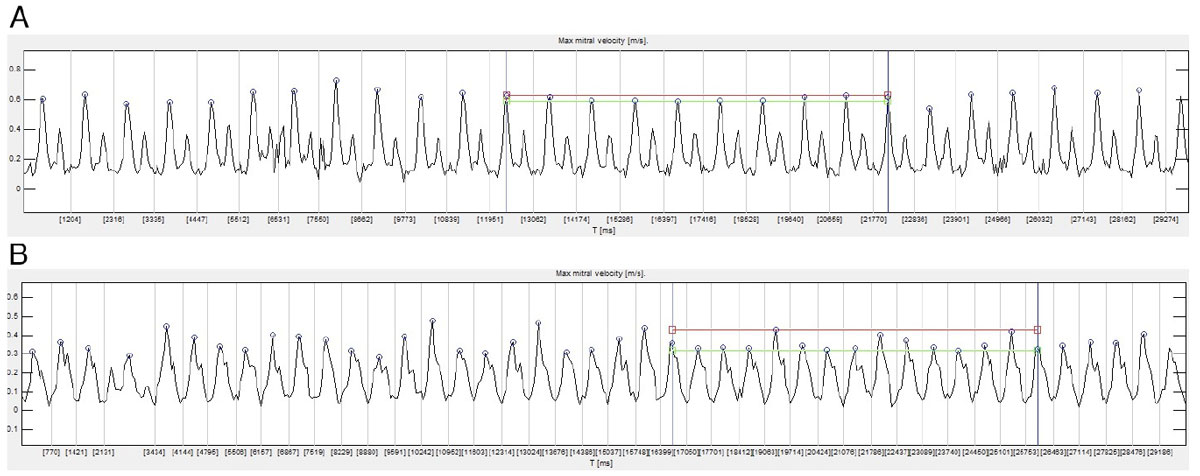
**Examples from two patients (A and B) showing RT-PC velocity data as analysed by the software plugin**. Each cardiac cycle is marked by vertical grey line and E-wave peak maximum velocity within each cardiac cycle is marked by a blue circle. Maximum and minimum peak velocity levels within that interval are shown as horizontal red and green lines, respectively. The vertical blue lines denote the 10-second interval showing the lowest velocity variation. Patient A had a variation of 7% and patient B was the patient with 20 mm pericardial effusion and a variation of 34%. Patient A had a heartrate of 54 beats/min and patient B had a heartrate of 88 beats/min. Due to a relatively high heart rate patient B had partial E and A-wave fusion.

